# The S-shaped association between dietary caffeine intake and severe headache or migraine: a cross-sectional study based on NHANES

**DOI:** 10.3389/fneur.2025.1517942

**Published:** 2025-05-14

**Authors:** Zhiqiang Liao, Aiqing Lin, Junjian Zeng, Yu Zou, YiXun Chen, Zhonghua Liu, Zhidong Zhou

**Affiliations:** ^1^Department of Anesthesiology, The Second Affiliated Hospital, Jiangxi Medical College, Nanchang University, Nanchang, Jiangxi, China; ^2^Jiangxi Province Key Laboratory of Anesthesiology, Nanchang, Jiangxi, China

**Keywords:** caffeine, severe headache, migraine, cross-sectional study, NHANES

## Abstract

**Background and purpose:**

Severe headache or migraine is a highly prevalent neurological disorder that significantly impacts the daily lives of those affected. Currently, there remains a debate regarding the relationship between daily caffeine intake and severe headache or migraine. This study aims to determine the relationship between caffeine intake and severe headache or migraine to inform dietary interventions.

**Materials and methods:**

The study utilized data from the National Health and Nutrition Examination Survey (NHANES) conducted between 1999 and 2004, involving a total of 5,234 participants aged 20–49 years. A multivariable logistic regression model was employed to examine the relationship between dietary caffeine intake and severe headache or migraine. A restricted cubic spline (RCS) regression model was used to explore potential dose–response relationships. A smoothed two-piece logistic regression model was applied to identify threshold associations between dietary caffeine intake and severe headache or migraine. Subgroup regression analyses were conducted to investigate whether the impact of dietary caffeine intake on severe headache or migraine varied among different subgroups.

**Results:**

Among the 5,234 participants, 26.69% (1,397/5234) had severe headache or migraine. Compared to individuals with lower caffeine intake (quartile [Q]1, ≤1.81 mg/d), those with higher caffeine intake in Q2 (1.81–45.48 mg/d), Q3 (45.48–125.95 mg/d), and Q4 (≥125.95 mg/d) had adjusted odds ratios for severe headache or migraine of 1.1487 (95% confidence interval [CI]: 0.9532–1.3845, *p* = 0.1454), 1.4370 (95% CI: 1.1921–1.7335, *p* = 0.001), and 1.5642 (95% CI: 1.2842–1.9067, *p* < 0.001), respectively. The relationship between dietary caffeine intake and severe headache or migraine in U.S. adults exhibits an S-shaped pattern, with a turning point at approximately 97.5 mg/d.

**Conclusion:**

These results suggest an S-shaped association between dietary caffeine intake and severe headache or migraine. Individuals who routinely consume caffeine in their diet should be vigilant about the risk of experiencing severe headaches or migraines.

## 1 Introduction

A study based on the Epic Cosmos national database in the United States has revealed that over the past 8 years, there has been a consistent but slight upward trend in the rate of emergency department visits for headaches, with an average visitation rate of 3.2% and a hospital admission rate of 4.1%. Notably, individuals under the age of 50 accounted for 66.2% of these visits (1). Migraine is classified by the World Health Organization (WHO) as the third most common disease globally and the sixth leading cause of years lost due to disability (2). Migraine is often characterized by unilateral throbbing pain in the brain, lasting from 4 to 72 h, and is accompanied by symptoms such as photophobia, nausea, and vomiting (3). Severe headaches or migraines increase both the cost of living and time expenses. The factors influencing migraine attacks are numerous and complex, including gut microbiota factors such as Bacteroides and Streptococcus, as well as dietary factors like alcohol and caffeine (4). In addition, recent research indicates that different types of micronutrients, such as vitamin B1 and vitamin C, are also associated with severe headaches or migraines (5, 6). This suggests that the types and dosages of dietary components can have varying impacts on severe pain or migraines. Caffeine, a naturally occurring methylxanthine, is considered the most widely consumed psychoactive substance in the world (7). In daily life, caffeine primarily comes from coffee beverages, but it is also found in smaller amounts in tea leaves, chocolate, energy drinks, and other sources. Due to its structural similarity to adenosine, caffeine can competitively bind to adenosine receptors (such as A1 and A2A receptors) in the body. Activation or inhibition of different types of adenosine receptors in the Trigeminovascular System (TVS) is thought to be closely related to the onset of migraines. Trigeminovascular neurons play a crucial role in receiving and transmitting pain signals, contributing significantly to the process of central and peripheral sensitization (8). Currently, there is limited and contradictory research on the relationship between dietary caffeine intake and severe headache or migraine. Previous studies have suggested that the intake of both dietary and medicinal caffeine appears to be a moderate risk factor for the onset of chronic headache across various headache types (9). A recent study from Japan has also revealed that caffeine intake is associated with an increase in the frequency and severity of migraine episodes in children and adolescents (10). However, a prospective cohort study indicated that there is no significant association between caffeine consumption from beverages and headache frequency, duration, or intensity (11). Therefore, in this study, we conducted a cross-sectional analysis based on data from the National Health and Nutrition Examination Survey (NHANES) from 1999 to 2004. The aim was to investigate the relationship between daily dietary caffeine intake and severe headache or migraine, with the hope of providing new evidence to guide dietary interventions for these conditions.

## 2 Methods

### 2.1 Study population

**Figure 1:**
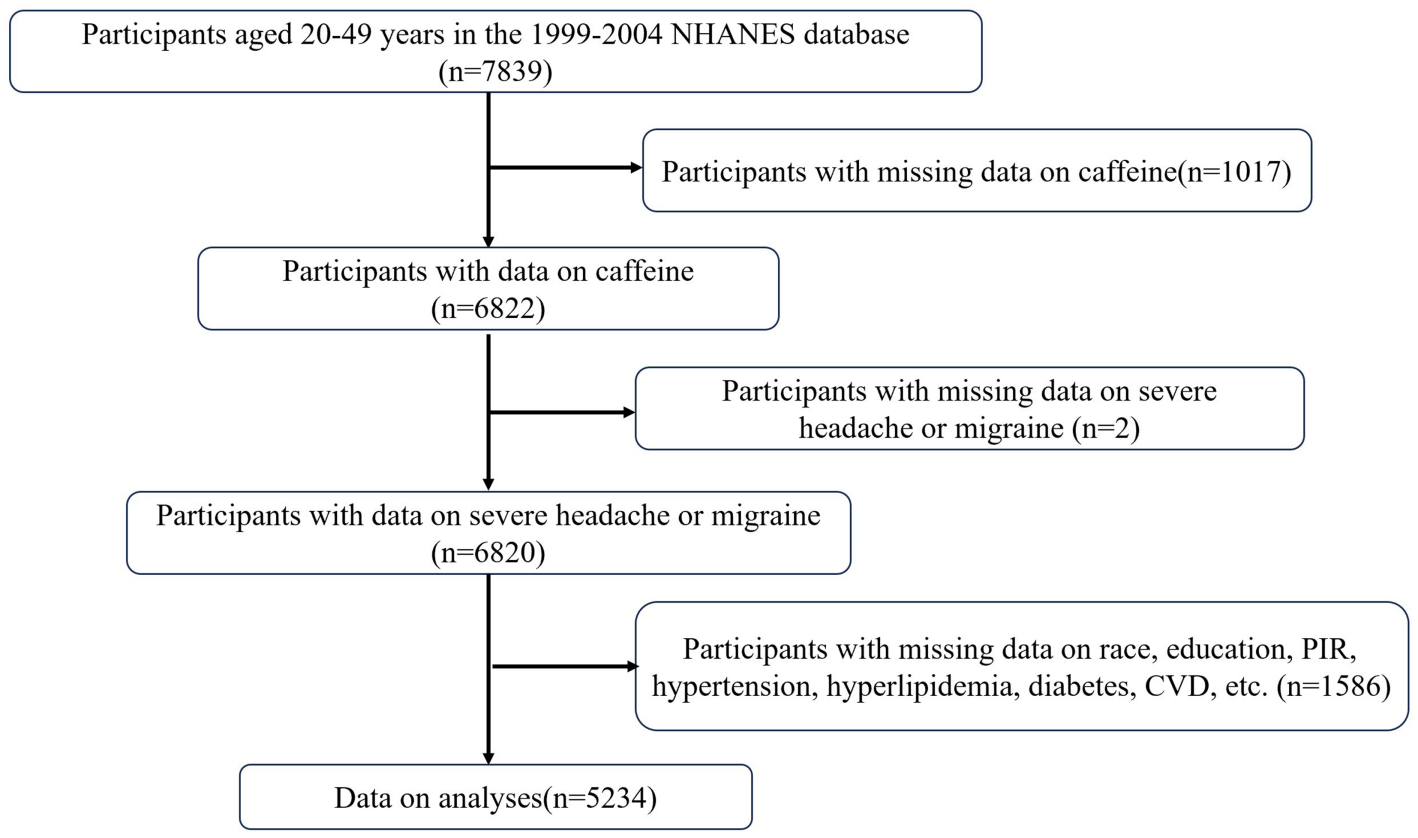
Flowchart of the study.

This study is based on the publicly available NHANES database from 1999 to 2004. The study protocol for NHANES was approved by the Ethics Review Board of the National Center for Health Statistics (Protocol #98–12, https://www.cdc.gov/nchs/nhanes/about/erb.html?CDC_AAref_Val). The NHANES research program is designed to assess the health and nutritional status of non-institutionalized Americans using a stratified, multistage probability survey. A total of 31,126 individuals were interviewed. From this group, participants aged 20–49 years (*n* = 7,839) were selected. Those with missing data on dietary caffeine intake (*n* = 1,017), history of severe headache or migraine (*n* = 2), and covariates (*n* = 1,586) were excluded. Therefore, this cross-sectional study analyzed a final sample of 5,234 NHANES participants from 1999 to 2004. Detailed information regarding participant recruitment is illustrated in Figure 1.

### 2.2 Dietary caffeine intake

Dietary caffeine intake (in milligrams) was based on NHANES dietary interviews and total nutrient intake data. Data collection involved two 24-h interviews. The first interview was conducted by professionally trained dietary interviewers at the Mobile Examination Center (MEC), followed by a second telephone interview 3–10 days later. In the NHANES 1999–2002 cycle, only the dietary data from the first 24-h recall interview were available. However, for the 2003–2004 cycle, data from two dietary recall interviews were available. To ensure the accuracy of the study, the mean of the non-missing data from the two interviews in the 2003–2004 cycle was used.

### 2.3 Definition of severe headache or migraine

According to the 1999–2004 NHANES pain questionnaire, severe headache or migraine was defined by the question: “Have you had a severe headache or migraine in the past 3 months?” Participants who responded affirmatively were classified as having severe headaches or migraines.

### 2.4 Other covariates

Based on clinical experience and existing literature, key potential covariates include gender (male or female), age (20–49 years), race/ethnicity (Mexican American, other Hispanic, non-Hispanic White, non-Hispanic Black, or other races), educational level (less than high school, high school, and more than high school), marital status (married, unmarried, widowed, separated, divorced, or living with a partner), family poverty income ratio (PIR) (<1.3, 1.3 ≤ PIR < 3.5, or ≥3.5), body mass index (BMI) (<18.9, 18.9 ≤ BMI < 24.9, 24.9 ≤ BMI < 30, or ≥30), smoking status (never, former, or current), alcohol consumption (never, former, or current), and physical activity level (none, moderate, vigorous), C-reactive protein (CRP). Diabetes was defined as meeting any of the following criteria: (1) a previous diagnosis of diabetes mellitus (DM) by a doctor; (2) hemoglobin A1c (glycated hemoglobin) level greater than 6.5%; (3) fasting blood glucose level ≥7.0 mmol/L; (4) use of diabetes medication or insulin. Hyperlipidemia was defined as having any of the following: total cholesterol ≥200 mg/dL, triglycerides ≥150 mg/dL, high-density lipoprotein (HDL) ≤ 40 mg/dL for males or ≤50 mg/dL for females, or low-density lipoprotein (LDL) ≥ 130 mg/dL. Additionally, individuals using lipid-lowering medications were also considered to have hyperlipidemia. The diagnosis of cardiovascular disease (CVD) was based on self-reported history of congestive heart failure, coronary heart disease, angina, heart attack, or stroke. Hypertension was defined as having an average systolic blood pressure (SBP) ≥ 130 mmHg or an average diastolic blood pressure (DBP) ≥ 80 mmHg, or having a diagnosis of hypertension along with the use of antihypertensive medication. The use of analgesic pain medications was defined as whether an individual has taken any prescription or over-the-counter pain relievers almost every day.

### 2.5 Statistical analyses

This study is a secondary analysis based on the publicly accessible NHANES database. Categorical variables are represented as percentages (%), while continuous variables are presented as means (standard deviation, SD). The Kruskal–Wallis rank-sum test and Pearson’s Chi-squared test were used to compare the baseline characteristics of continuous and categorical variables, respectively. Additionally, multivariable logistic regression analysis was employed to investigate the relationship between caffeine intake and severe headache or migraine. Based on previous literature and clinical experience, different models included relevant adjustment variables. Model 1 controlled for gender, age, race/ethnicity, educational level, marital status, and PIR. Model 2 additionally controlled for BMI, physical activity, smoking status, and alcohol consumption. Model 3 further adjusted for diabetes, hypertension, hyperlipidemia, CVD, CRP levels, and the use of analgesic pain medications. Based on Model 3, regression analysis was conducted using smoothed curve fitting and restricted cubic splines (RCS), with three knots placed at specific percentiles of dietary caffeine intake. This analysis aimed to assess both linear and dose–response curves between dietary caffeine intake and severe headache or migraine. Additionally, the inflection point for non-linear relationships was calculated using a recursive algorithm and two-phase linear regression model. Finally, subgroup interaction regression analyses were performed, stratifying by caffeine intake quartiles and considering variables such as gender, PIR, BMI, physical activity, and alcohol and smoking status. Statistical analyses for this study were conducted using DecisionLink 1.0, with a *p*-value of <0.05 considered to indicate statistical significance.

## 3 Results

### 3.1 Baseline characteristics

The average age of all participants was 34.02 years, with 45.36% male and 54.64% female. The mean caffeine intake was 116.47 mg, and the prevalence of migraines was 26.68%. Baseline characteristics according to quartiles of caffeine intake are shown in Table 1. There were statistically significant differences (*p* < 0.05) among different dietary caffeine intake groups in terms of age, gender, race, education level, marital status, PIR, smoking status, alcohol consumption, hypertension, physical activity, CRP levels, use of analgesic pain medications, and prevalence of severe headache or migraine. Participants in the high caffeine intake group (Group 4) were more likely to be older, male, non-Hispanic White, have a high school education or above, be married/widowed/divorced, have a PIR ≥ 3.5, have a BMI between 24.9 and <30, be former or current smokers, be former or current drinkers, have hypertension, and engage in moderate physical activity.

**Table 1:** Characteristics of the study population from NHANES 1999–2004.

Characteristic	Total	Caffeine intake (mg/day)				*P*-value
		Q1	Q2	Q3	Q4	
No.	5,234	1,318	1,299	1,309	1,308	
Caffeine, mean ± SD	116.47 ± 120.31	1.81 ± 2.92	45.48 ± 18.40	125.95 ± 30.35	293.01 ± 84.49	
Age (years, mean ± SD)	34.02 ± 8.67	32.44 ± 8.51	32.62 ± 8.62	34.20 ± 8.68	36.83 ± 8.14	<0.001
Sex, *n* (%)						<0.001
Male	2,374 (45.36)	518 (39.30)	538 (41.42)	615 (46.98)	703 (53.75)	
Female	2,860 (54.64)	800 (60.70)	761 (58.58)	694 (53.02)	605 (46.25)	
Race, *n* (%)						<0.001
Mexican American	1,344 (25.68)	361 (27.39)	405 (31.18)	335 (25.59)	243 (18.58)	
Other Hispanic	271 (5.18)	55 (4.17)	88 (6.77)	73 (5.58)	55 (4.20)	
Non-Hispanic White	2,349 (44.88)	437 (33.16)	471 (36.26)	602 (45.99)	839 (64.14)	
Non-Hispanic Black	1,073 (20.50)	413 (31.34)	294 (22.63)	241 (18.41)	125 (9.56)	
Other Race	197 (3.76)	52 (3.95)	41 (3.16)	58 (4.43)	46 (3.52)	
Educational level, *n* (%)						<0.001
Less than high school	1,340 (25.60)	376 (28.53)	383 (29.48)	330 (25.21)	251 (19.19)	
High school	1,237 (23.63)	288 (21.85)	310 (23.86)	295 (22.54)	344 (26.30)	
More than high school	2,657 (50.76)	654 (49.62)	606 (46.65)	684 (52.25)	713 (54.51)	
Marital status, *n* (%)						<0.001
Married	2,852 (54.49)	670 (50.83)	693 (53.35)	719 (54.93)	770 (58.87)	
Widowed	33 (0.63)	7 (0.53)	7 (0.54)	9 (0.69)	10 (0.76)	
Divorced	349 (6.67)	77 (5.84)	75 (5.77)	85 (6.49)	112 (8.56)	
Separated	204 (3.90)	53 (4.02)	50 (3.85)	55(4.20)	46 (3.52)	
Never married	1,334 (25.49)	396 (30.05)	362 (27.87)	323 (24.68)	253 (19.34)	
Living with partner	462 (8.83)	115 (8.73)	112 (8.62)	118 (9.01)	117 (8.94)	
PIR, *n* (%)						<0.001
<1.3	1,501 (28.68)	440 (33.38)	428 (32.95)	353 (26.97)	280 (21.41)	
1.3–3.5	1991 (38.04)	491 (37.25)	534 (41.11)	499 (38.12)	467 (35.70)	
≥3.5	1742 (33.28)	387 (29.36)	337 (25.94)	457 (34.91)	561 (42.89)	
BMI, *n* (%)						0.708
<18.4	85 (1.62)	22 (1.67)	18 (1.39)	25 (1.91)	20 (1.53)	
18.4 ≥ BMI<24.9	1,683 (32.16)	441 (33.46)	412 (31.72)	411 (31.40)	419 (32.03)	
24.9 ≥ BMI<30	1760 (33.63)	437 (33.16)	420 (32.33)	444 (33.92)	459 (35.09)	
≥30	1706 (32.59)	418 (31.71)	449 (34.57)	429 (32.77)	410 (31.35)	
Hyperlipidemia, *n* (%)						0.254
No	1719 (32.84)	447 (33.92)	398 (30.64)	442 (33.77)	432 (33.03)	
Yes	3,515 (67.16)	871 (66.08)	901 (69.36)	867 (66.23)	876 (66.97%)	
Alcohol status, *n* (%)						<0.001
None	1,482 (28.31)	502 (38.09)	413 (31.79)	344 (26.28)	223 (17.05)	
Former	332 (6.34)	74 (5.61)	62 (4.77)	91 (6.95)	105 (8.03)	
Current	3,420 (65.34)	742 (56.30)	824 (63.43)	874 (66.77)	980 (74.92)	
Smoking status, *n* (%)						<0.001
None	3,029 (57.87)	889 (67.45)	819 (63.05)	723 (55.23)	598 (45.72)	
Former	851 (16.26)	172 (13.05)	202 (15.55)	231 (17.65)	246 (18.81)	
Current	1,354 (25.87)	257 (19.50)	278 (21.40)	355 (27.12)	464 (35.47)	
Hypertension, *n* (%)						<0.001
No	3,420 (65.34)	883 (67.00)	896 (68.98)	843 (64.40)	798 (61.01)	
Yes	1814 (34.66)	435 (33.00)	403 (31.02)	466 (35.60)	510 (38.99)	
Diabetes, *n* (%)						0.112
No	4,993 (95.40)	1,246 (94.54)	1,252 (96.38)	1,253 (95.72)	1,242 (94.95)	
Yes	241 (4.60)	72 (5.46)	47 (3.62)	56 (4.28)	66 (5.05)	
CVD, *n* (%)						0.997
No	5,130 (98.01)	1,291 (97.95)	1,274 (98.08)	1,283 (98.01)	1,282 (98.01)	
Yes	104 (1.99)	27 (2.05)	25 (1.92)	26 (1.99)	26 (1.99)	
Physical activity, *n* (%)						<0.001
None	1879 (35.90)	478 (36.27)	519 (39.95)	462 (35.29)	420 (32.11)	
Moderate	2,591 (49.50)	624 (47.34)	600 (46.19)	676 (51.64)	691 (52.83)	
Vigorous	764 (14.60)	216 (16.39)	180 (13.86)	171 (13.06)	197 (15.06)	
Drug, *n* (%)						<0.001
No	4,510 (86.17)	1,169(88.69)	1,146 (88.22)	1,133 (86.55)	1,062 (81.19)	
Yes	724 (13.83)	149 (11.31)	153 (11.78)	176 (13.45)	246 (18.81)	
CRP, Mean ± SD	0.44 ± 0.78	0.47 ± 0.73	0.44 ± 0.66	0.49 ± 1.08	0.37 ± 0.57	0.002
Migraine or severe headache, *n* (%)						0.027
No	3,837 (73.31)	1,002 (76.02)	961 (73.98)	940 (71.81)	934 (71.41)	
Yes	1,397 (26.69)	316 (23.98)	338 (26.02)	369 (28.19)	374 (28.59)	

PIR, poverty income ratio; BMI, body mass index; CVD, cardiovascular disease; Drug, analgesic pain medications; CRP, C-reactive protein.

### 3.2 Association of dietary caffeine intake with severe headache or migraine

In this study, we constructed three models to analyze the relationship between dietary caffeine intake and severe headache or migraine. The results are presented in Table 2. When dietary caffeine intake was examined as a continuous variable, a positive association between caffeine intake and severe headache or migraine was observed across all three models, which adjusted for relevant covariates (Model 3: OR = 1.0013, 95% CI: 1.0007–1.0018, *p* < 0.0001). Further analysis by quartiles of dietary caffeine intake, with the same adjustments, revealed that compared to the lowest caffeine intake group (Q1), those in higher intake groups (Q2, Q3, Q4) showed adjusted odds ratios (ORs) for severe headache or migraine in Model 3 of 1.1487 (95% CI: 0.9532–1.3845, *p* = 0.1454), 1.4370 (95% CI: 1.1921–1.7335, *p* = 0.0001), and 1.5642 (95% CI: 1.2842–1.9067, *p* < 0.0001), respectively. These results suggest a potential non-linear relationship between dietary caffeine intake and severe headache or migraine.

**Table 2:** Association of dietary caffeine intake with severe headache or migraine among NHANES survey participants 1999–2004.

Variable	Model 1		Model 2		Model 3	
	OR (95% CI)	*P*-value	OR (95% CI)	*P*-value	OR (95% CI)	*P*-value
Caffeine	1.0006 (1.0001, 1.0011)	0.0134	1.0014 (1.0008, 1.0019)	<0.0001	1.0013 (1.0007, 1.0018)	<0.0001
Caffeine (quartile)						
Q1	Reference		Reference		Reference	
Q2	1.1282 (0.9399, 1.3546)	0.1955	1.1332 (0.9433, 1.3617)	0.1817	1.1487 (0.9532, 1.3845)	0.1454
Q3	1.4033 (1.1696, 1.6846)	0.0003	1.3979 (1.1634, 1.6808)	0.0004	1.4370 (1.1921, 1.7335)	0.0001
Q4	1.5700 (1.2977, 1.9009)	<0.0001	1.5523 (1.2793, 1.8850)	<0.0001	1.5642 (1.2842, 1.9067)	<0.0001
*P* for trend	<0.0001		<0.0001		<0.0001	

95%CI: confidence interval.

Model l: adjust for sex, age, race, educational level, marital status, PIR.

Model 2: adjust for sex, age, race, educational level, marital status, PIR, BMI, alcohol, smoking, physical activity.

Model 3: adjust for sex, age, race, educational level, marital status, PIR, BMI, alcohol, smoking, physical activity, hyperlipidemia, hypertension, diabetes, analgesic pain medications, CVD, CRP.

### 3.3 Smooth curve fitting analysis, restricted cubic spline regression analysis and threshold analysis

**Figure 2:**
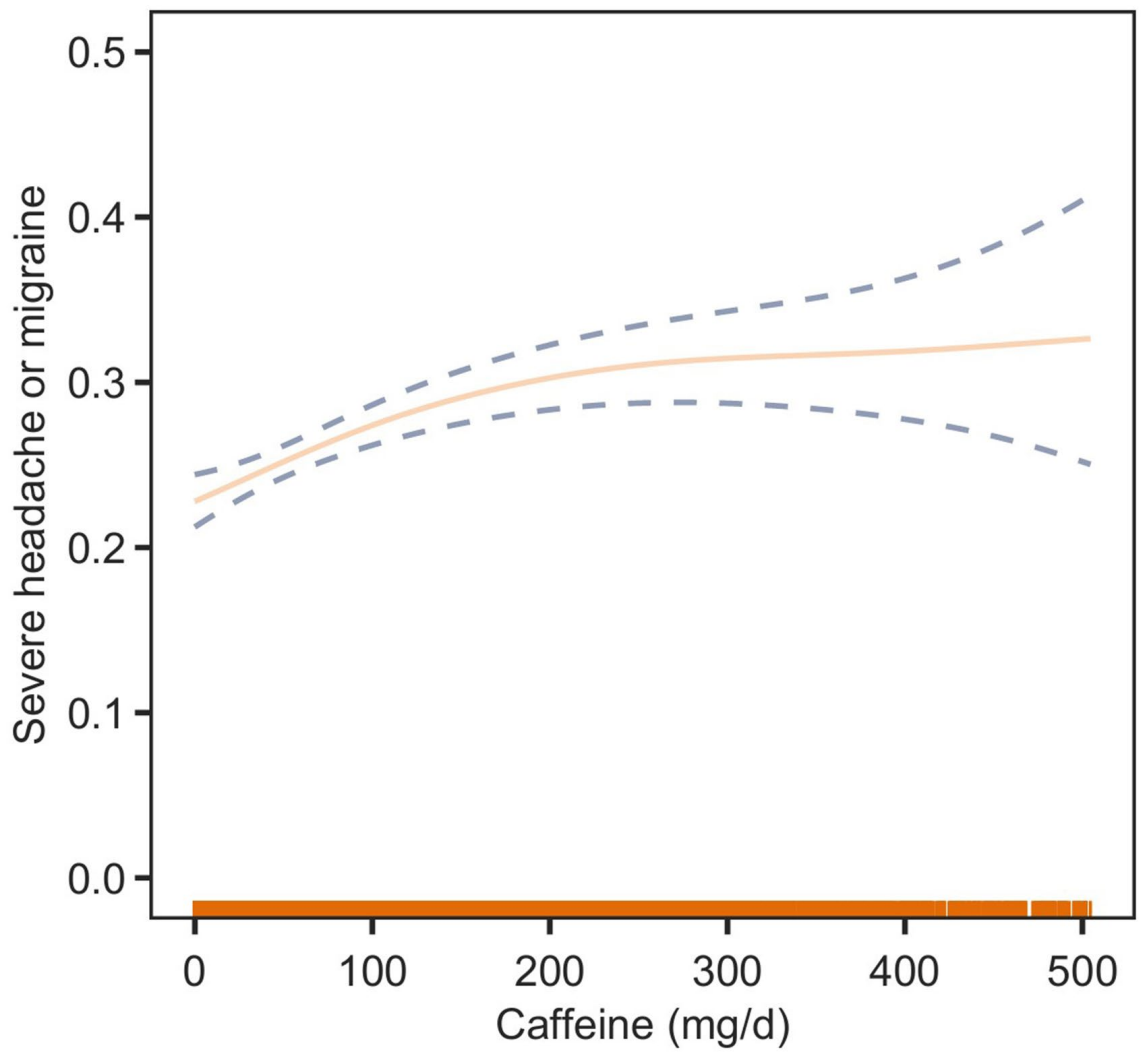
Association between dietary caffeine intake and severe headache or migraine: smooth curve fitting plot. The solid line represents the predicted values, and the dashed lines represent the 95% confidence intervals.

**Figure 3:**
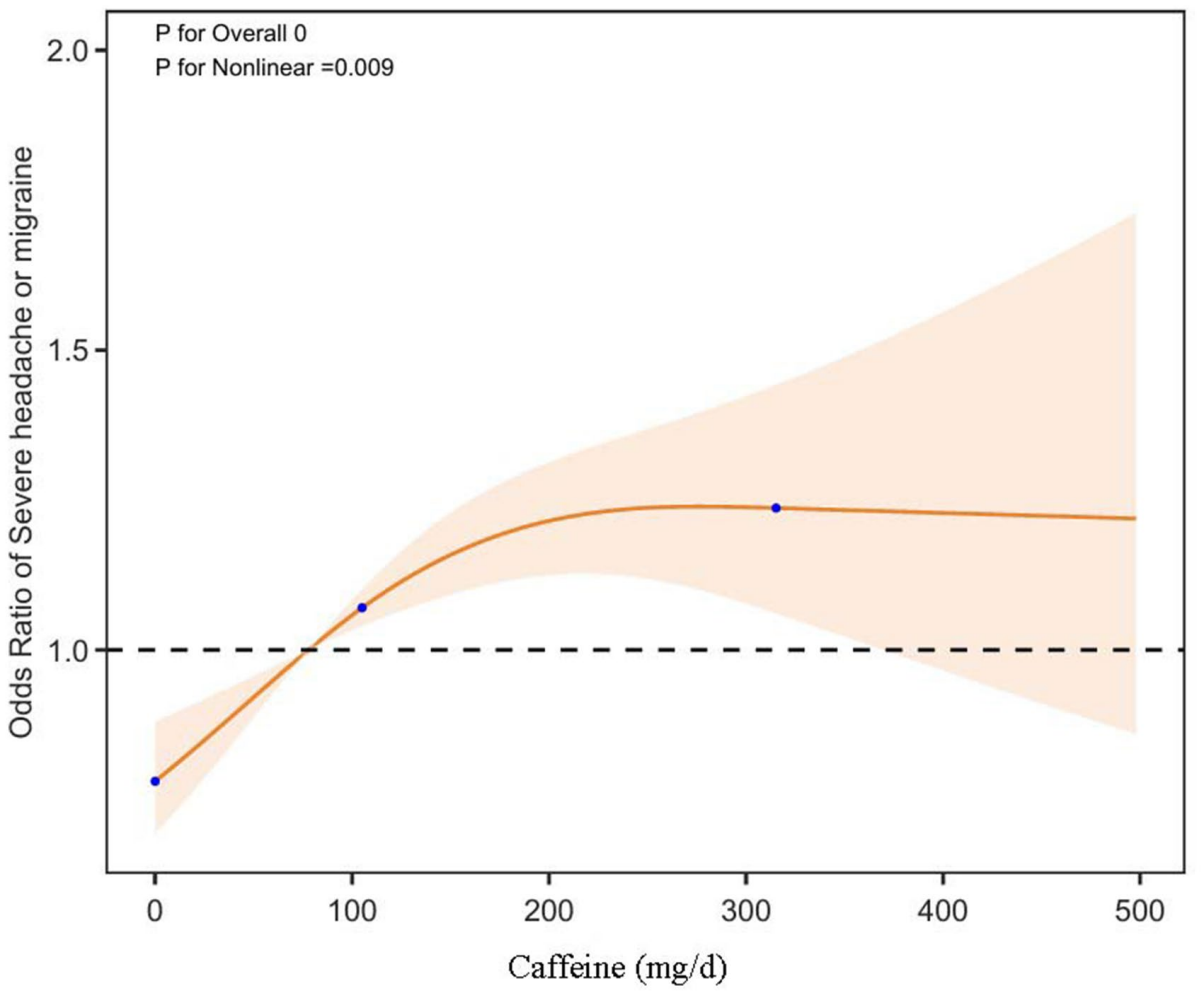
Dose-effect relationship between dietary caffeine intake and severe headache or migraine: restricted cubic spline regression analysis (RCS) plot. The solid line represents the predicted values, and the shaded orange area indicates the 95% confidence intervals.

To further elucidate the relationship between dietary caffeine intake and severe headache or migraine, we generated a smooth curve fitting plot (Figure 2) and a restricted cubic spline plot (RCS) (Figure 3), both adjusted for covariates in Model 3. Both Figures 2, 3 demonstrate a non-linear association between dietary caffeine intake and severe headache or migraine, which may explain the lack of statistical significance in some analyses. More importantly, in the threshold analysis, participants with caffeine intake below 97.5 mg/d had an odds ratio of 1.0038 (95% CI: 1.0019–1.0058, *p* = 0.0001) for developing severe headache or migraine, as shown in Table 3. This indicates that each additional milligram of dietary caffeine intake per day increases the risk of severe headache or migraine by 0.38%. For individuals with a daily caffeine intake of ≥97.5 mg/d, there was no significant association between dietary caffeine intake and severe headache or migraine (Table 3).

**Table 3:** Threshold effect analysis of the relationship of dietary caffeine intake with severe headache or migraine.

Threshold of caffeine intake	Adjusted model	
	OR (95%CI)	*P*-value
<97.5 (mg/d)	1.0038 (1.0019, 1.0058)	0.0001
≥97.5 (mg/d)	1.0003 (0.9994, 1.0011)	0.5240
Log-likelihood ratio test		0.0055

### 3.4 Subgroup interactive regression analysis

Subgroup interaction regression analysis was employed to stratify participants based on gender, PIR, BMI, physical activity, and smoking and drinking status. The impact of dietary caffeine intake on the risk of severe headaches or migraines was evaluated within each subgroup. In the male population, the incidence of severe headaches or migraines significantly increased with a higher intake of caffeine (Q2: OR = 2.81, 95% CI: 2.1, 3.78; Q3: OR = 2.42, 95% CI: 1.88, 3.13; Q4: OR = 2.15, 95% CI: 1.68, 2.75). A similar trend was observed in females, albeit with a slightly lower magnitude of increase (Q2: OR = 2.76, 95% CI: 2.1, 3.67; Q3: OR = 2.42, 95% CI: 1.88, 3.13; Q4: OR = 2.15, 95% CI: 1.68, 2.75). However, the interaction between gender and caffeine intake was not significant (interaction *p* = 0.21 > 0.05), indicating no substantial interaction effect between gender and the amount of caffeine consumed. No significant effects were found in the other subgroups regarding the correlation between dietary caffeine intake and the occurrence of severe headaches or migraines (Supplementary Figure S1).

## 4 Discussion

This cross-sectional study aimed to investigate the relationship between dietary caffeine intake and the occurrence of severe headaches or migraines. After accounting for potential confounding factors, the results indicated that, among the American population aged 20–49, the relationship between dietary caffeine intake and the incidence of severe headaches or migraines follows an S-shaped pattern, with an inflection point around 97.5 mg/d.

The relationship between dietary status and the incidence of severe or chronic migraines has been documented, with a healthy dietary state possibly linked to lower occurrence rates and delayed progression of headaches or migraines. For instance, a cross-sectional study from the Netherlands indicated that 35.6% of patients reported alcoholic beverages as a trigger for migraine attacks, with red wine being the most frequently identified precipitant among alcoholic drinks (12). The incidence of migraines in individuals who skip breakfast is significantly higher than in those who consume breakfast (13). Statistical research indicates that coffee is the primary source of dietary caffeine for consumers, with about 85% of Americans drinking at least one caffeinated beverage per day (14). Caffeine, with its structure as 1,3,7-trimethylxanthine, has an average half-life of 5 h and can act as a non-selective antagonist for adenosine receptors, which are G protein-coupled receptors (GPCRs) in the human body (15). Adenosine receptors include four types: A1, A2A, A2B, and A3. It is currently recognized that the expression of adenosine A1 receptors is present in the trigeminal ganglion (TG) and the caudal trigeminal nucleus (TNC) within the TVS. Activation of A1 receptors can inhibit migraine attacks by suppressing trigeminal nerve firing and reducing the release of Calcitonin Gene-Related Peptide (CGRP), consequently inhibiting the TVS. On the other hand, activation of A2 receptors leads to the dilation of extracerebral vessels, subsequently activating the TVS and resulting in the onset of migraines (16). It is important to note that caffeine has dose-dependent biological effects on the nervous system (17). Research indicates that moderate doses of caffeine (e.g., 50 μM) can effectively block the A1 receptor, enhancing neuronal excitability and synaptic transmission, thus exerting both neurostimulatory and neuroprotective effects (18). High doses of caffeine (e.g., over 100 μM or higher) may have toxic effects on the nervous system, and these toxic effects are associated with adenosine receptors. For example, after high-dose caffeine completely blocks the A1 receptor, it induces neuronal excitability toxicity (19), while blocking both A1 and A2A receptors leads to disruption of synaptic transmission (such as abnormally enhanced glutamatergic signaling) and energy metabolism crisis (inhibiting adenosine through A1 receptors reduces neuronal energy consumption) (18). Moreover, chronic high-dose caffeine may also trigger compensatory upregulation of A2A receptors, leading to neuroinflammation or abnormal neural circuit remodeling (20). Furthermore, high doses of caffeine affect the nervous system through other mechanisms, such as inhibiting phosphodiesterases (PDEs), which increases intracellular cAMP levels and leads to abnormal neuronal signaling (21), and binding to ryanodine receptors, which promotes intracellular calcium release and causes neurotoxicity or cell apoptosis (22). Thus, varying doses of caffeine exert distinct biological effects on the nervous system through mechanisms like adenosine receptor modulation. A case–control study found that individuals who consume 1–2 cups of coffee daily have a 1.439 times higher risk of developing migraines compared to those who do not drink coffee (one cup of coffee contains approximately 80 mg of caffeine) (23). This aligns with our findings, which suggest that increasing caffeine consumption significantly raises the risk of severe headaches or migraines. Concurrently, the biological effects of caffeine are tightly linked to its metabolism (24). Caffeine taken orally is absorbed at a rate of 99% within 45 min and is mainly metabolized by the liver’s cytochrome P450 enzyme system, particularly CYP1A2, resulting in the production of metabolites like paraxanthine (84%), theobromine (12%), and theophylline (4%) (25). Furthermore, the activity of CYP1A2 differs across various age groups, which causes variations in caffeine metabolism at different life stages (26). For instance, the half-life of caffeine in neonates can extend up to 80 h, while in healthy adults aged 20–49, liver enzyme activity peaks, resulting in the fastest caffeine metabolism within 3–7 h. In elderly individuals, enzyme activity decreases due to factors such as reduced hepatic blood flow, prolonging the half-life to 8–10 h (24). As a result, with the same caffeine intake, circulating caffeine concentrations vary across different age groups, leading to differences in peak caffeine levels and duration of exposure. In the elderly, reduced CYP1A2 activity results in sustained high levels of caffeine in the body, which may intensify the antagonistic effects on adenosine receptors (A₁/A₂A). Furthermore, the increased sensitivity of A₂A receptors in older adults makes even low doses of caffeine potent enough to enhance vasoconstriction, triggering the TVS and leading to headaches (27). In adults, with relatively stable CYP1A2 activity, genetic polymorphism in the CYP1A2 gene, along with factors like smoking and certain medications, are key considerations in caffeine metabolism (28). Our study, focusing on U.S. adults aged 20–49, discovered a non-linear relationship between dietary caffeine and severe headaches or migraines after controlling for the potential influence of confounding factors. This indicates that individuals with severe headaches or migraines should manage their caffeine intake to reduce caffeine concentrations in the body, thereby decreasing the occurrence of headaches or migraines.

Furthermore, varying doses of caffeine may influence the onset and progression of headache and other related neurological disorders by affecting sleep quality and the body’s electrolyte balance. The effect of caffeine on sleep quality is bidirectional (29). Moderate caffeine intake can slightly increase alertness and promote wakefulness through the blockade of adenosine receptors (A1/A2A), while high doses of caffeine, by elevating cortisol levels and activating the stress system, may disrupt sleep architecture (e.g., prolonging sleep latency, reducing slow-wave sleep, shortening total sleep time, and worsening perceived sleep quality) (30). A case-crossover randomized trial demonstrated that each additional cup of coffee, averaging 95 mg of caffeine, was associated with a 14-min reduction in nightly sleep (95% CI 10–18 min, *p* < 0.001) (31). Additionally, a recent randomized controlled trial showed that caffeine impairs recovery after sleep deprivation and prevents improvements in sleep quality. Specifically, in adults following sleep deprivation, consuming 2.5 mg/kg caffeine significantly reduced total sleep time during recovery by 30.2 ± 8.2 min compared to the placebo group (*p* = 0.02), and decreased the duration of deep sleep (N3 stage) by 35.6 ± 23.2 min (*p* < 0.01) (32). At the same time, the decline in sleep quality can reduce the pain threshold by accumulating adenosine, inhibit the analgesic effects of 5-HT (serotonin), and enhance the sensitivity of the TVS, all of which can trigger or promote the occurrence of headache-related diseases (33). In other words, the impact of caffeine intake on headaches and other neurological disorders may, in part, be mediated through a reduction in sleep quality (15). In addition, caffeine also mediates the occurrence of headaches or migraines by influencing the body’s water-electrolyte balance. Caffeine exerts a diuretic effect through mechanisms such as antagonizing A1 or A2A receptors in the proximal tubule cells of the kidney, inhibiting Na^+^/K^+^-ATPase in renal tubules, and disrupting antidiuretic hormone (ADH) secretion (34). Research indicates that after chewing gum with a high caffeine dose, healthy adults showed a 67% increase in urine volume, and the renal clearance rates of sodium and calcium increased by 61 and 77%, respectively (35). The body’s internal balance can lead to electrolyte disturbances from the urinary excretion of potassium, magnesium, and calcium, thus affecting neural electrical activity in the brain and inducing oxidative stress in the brainstem. These changes are strongly associated with the pathophysiology of neurological disorders like headaches (36).

As previously mentioned, dietary caffeine plays a vital role in the onset and progression of neurological disorders such as severe headaches or migraines. In our study, we found that dietary caffeine intake exhibits an S-shaped relationship with severe headaches or migraines, suggesting that within a certain range, caffeine consumption may be detrimental to the prevention of these conditions. Specifically, for individuals with dietary caffeine intake below 97.5 mg/d, the likelihood of experiencing severe headaches or migraines increases as caffeine intake rises. Our study has several strengths. First, compared to previous studies of a similar nature, we have identified and further explored the non-linear relationship between dietary caffeine intake and severe headaches or migraines. Second, we treated the target variable (dietary caffeine intake) as both a continuous and a categorical variable, aiming to maximize the reliability of our findings. Additionally, we minimized residual confounding factors by employing rigorous statistical adjustments. Lastly, the NHANES database, recognized internationally as a high-quality resource, enhances the reliability and generalizability of our study results.

This study has several limitations. First, due to the inherent constraints of a cross-sectional design, we are unable to establish a causal relationship between dietary caffeine intake and severe headaches or migraines. Second, using data from the NHANES database, we could not distinguish whether individuals were habitual coffee drinkers, which prevents us from exploring further the relationship between dietary caffeine and other types of headaches, such as withdrawal headaches. Additionally, NHANES collects dietary caffeine intake data using a 24-h recall method, which is susceptible to recall bias. Lastly, the findings are based on adults aged 20–49 in the United States, and further research is needed to determine whether these results can be generalized to other populations. Furthermore, due to the limitations of the database, we are currently unable to clarify the role of CYP1A2 gene polymorphism in the relationship between caffeine intake and neurological disorders such as severe headaches or migraines in the adult population. Future research may further explore this aspect.

## 5 Conclusion

The association between dietary caffeine intake and severe headaches or migraines among U.S. adults aged 20–49 exhibits an S-shaped pattern, with a potential inflection point around 97.5 mg/d. These findings highlight the importance of considering the relationship between dietary caffeine consumption and the risk of severe headaches or migraines.

## Data Availability

Publicly available datasets were analyzed in this study. This data can be found here: https://www.cdc.gov/nchs/nhanes.

## References

[1] Gottlieb M Moyer E Bernard K. Epidemiology of headache presentations to United States emergency departments from 2016 to 2023. Am J Emerg Med. 85:1–6. doi: 10.1016/j.ajem.2024.08.013, PMID: 39141930

[2] GBD. Headache collaborators, global, regional, and national burden of migraine and tension-type headache, 1990-2016: a systematic analysis for the global burden of disease study 2016. Lancet Neurol. (2016) 17:954–76. doi: 10.1016/S1474-4422(18)30322-3, PMID: 30353868 PMC6191530

[3] International Classification of Headache Disorders, 3rd Edition (ICHD-3). Headache classification Committee of the International Headache Society (IHS) the international classification of headache disorders, 3rd edition. Cephalalgia. (2018) 38:1–211. doi: 10.1177/0333102417738202, PMID: 29368949

[4] Gazerani P Papetti L Dalkara T Cook CL Webster C Bai J. The brain, the eating plate, and the gut microbiome: Partners in Migraine Pathogenesis. Nutrients. (2024) 16:2222. doi: 10.3390/nu16142222, PMID: 39064664 PMC11280178

[5] Li D Guo Y Xia M Zhang J Zang W. Dietary intake of thiamine and riboflavin in relation to severe headache or migraine: a cross-sectional survey. Headache. (2022) 62:1133–42. doi: 10.1111/head.14384, PMID: 36047917

[6] Zheng Y Jin J Wei C Huang C. Association of dietary vitamin C consumption with severe headache or migraine among adults: a cross-sectional study of NHANES 1999-2004. Front Nutr. (2024) 11:1412031. doi: 10.3389/fnut.2024.1412031, PMID: 38962437 PMC11221565

[7] Israelsen IME Westgate CSJ Kamp-Jensen C Jensen RH Eftekhari S. Effects of caffeine on intracranial pressure and pain perception in freely moving rats. Headache. (2023) 63:1220–31. doi: 10.1111/head.14634, PMID: 37796087

[8] Iyengar S Johnson KW Ossipov MH Aurora SK. CGRP and the trigeminal system in migraine. Headache. (2019) 59:659–81. doi: 10.1111/head.13529, PMID: 30982963 PMC6593989

[9] Gadoth N Hering-Hanit R. Caffeine as a risk factor for chronic daily headache: a population-based study. Neurology. (2005) 65:180. doi: 10.1212/WNL.65.1.180, PMID: 16009928

[10] Hikita T Goda H Ogawa Y Kudo T Ito K. Caffeine consumption as a risk factor for childhood and adolescence migraine. Pediatr Int. (2023) 65:e15429. doi: 10.1111/ped.15429, PMID: 36461769

[11] Mittleman MR Mostofsky E Vgontzas A Bertisch SM. Habitual caffeinated beverage consumption and headaches among adults with episodic migraine: a prospective cohort study, headache: the journal of head and face. Pain. (2024) 64:299–305. doi: 10.1111/head.14673, PMID: 38318677 PMC10954400

[12] Onderwater GLJ van Oosterhout WPJ Schoonman GG Ferrari MD Terwindt GM. Alcoholic beverages as trigger factor and the effect on alcohol consumption behavior in patients with migraine. Eur J Neurol. (2019) 26:588–95. doi: 10.1111/ene.13861, PMID: 30565341

[13] Molarius A Tegelberg Å Öhrvik J. Socio-economic factors, lifestyle, and headache disorders — a population-based study in Sweden, headache: the journal of head and face. Pain. (2008) 48:1426–37. doi: 10.1111/j.1526-4610.2008.01178.x, PMID: 18624712

[14] Mitchell DC Knight CA Hockenberry J Teplansky R Hartman TJ. Beverage caffeine intakes in the U.S. Food Chem Toxicol. (2014) 63:136–42. doi: 10.1016/j.fct.2013.10.042, PMID: 24189158

[15] Charles A. The role of caffeine in headache disorders. Curr Opin Neurol. (2024) 37:289–94. doi: 10.1097/WCO.0000000000001249, PMID: 38327229

[16] Thuraiaiyah J Kokoti L Al-Karagholi MA-M Ashina M. Involvement of adenosine signaling pathway in migraine pathophysiology: a systematic review of preclinical studies. J Headache Pain. (2022) 23:43. doi: 10.1186/s10194-022-01412-0, PMID: 35382738 PMC8981838

[17] Pohanka M. The perspective of caffeine and caffeine derived compounds in therapy. Bratisl Lek Listy. (2015) 116:520–30. doi: 10.4149/BLL_2015_106, PMID: 26435014

[18] Kerkhofs A Xavier AC da Silva BS Canas PM Idema S Baayen JC. Caffeine controls glutamatergic synaptic transmission and pyramidal neuron excitability in human neocortex. Front Pharmacol. (2017) 8:899. doi: 10.3389/fphar.2017.00899, PMID: 29354052 PMC5758559

[19] Dunwiddie TV Masino SA. The role and regulation of adenosine in the central nervous system. Annu Rev Neurosci. (2001) 24:31–55. doi: 10.1146/annurev.neuro.24.1.31, PMID: 11283304

[20] Cunha RA. How does adenosine control neuronal dysfunction and neurodegeneration? J Neurochem. (2016) 139:1019–55. doi: 10.1111/jnc.13724 27365148

[21] Gupta A Pandey AN Sharma A Tiwari M Yadav PK Yadav AK. Cyclic nucleotide phosphodiesterase inhibitors: possible therapeutic drugs for female fertility regulation. Eur J Pharmacol. (2020) 883:173293. doi: 10.1016/j.ejphar.2020.173293, PMID: 32663542

[22] McPherson PS Kim YK Valdivia H Knudson CM Takekura H Franzini-Armstrong C. The brain ryanodine receptor: a caffeine-sensitive calcium release channel. Neuron. (1991) 7:17–25. doi: 10.1016/0896-6273(91)90070-G, PMID: 1648939

[23] Cha C Kim O Pang Y Jeong H Lee JE Lee H. Migraine incidence and coffee consumption among child-bearing age women: the Korea nurses’ health study. Sci Rep. (2024) 14:12760. doi: 10.1038/s41598-024-53302-x, PMID: 38834559 PMC11150557

[24] Nehlig A. Interindividual differences in caffeine metabolism and factors driving caffeine consumption. Pharmacol Rev. (2018) 70:384–411. doi: 10.1124/pr.117.014407, PMID: 29514871

[25] Zheng J Chen H Yang Q Zhou Z Yang C Huang J. Association of coffee consumption and caffeine metabolism with arrhythmias and cardiac morphology: an observational, genetic, and Mendelian randomization study. Heart Rhythm. (2024) S1547-5271:03631–2. doi: 10.1016/j.hrthm.2024.11.047, PMID: 39613205

[26] Zhang H Speakman JR. The complexity of coffee and its impact on metabolism. J Endocrinol. (2024) 262:e240075. doi: 10.1530/JOE-24-0075, PMID: 38885075

[27] Nowaczewska M Wiciński M Kaźmierczak W. The ambiguous role of caffeine in migraine headache: from trigger to treatment. Nutrients. (2020) 12:2259. doi: 10.3390/nu12082259, PMID: 32731623 PMC7468766

[28] Low JJ-L Tan BJ-W Yi L-X Zhou Z-D Tan E-K. Genetic susceptibility to caffeine intake and metabolism: a systematic review. J Transl Med. (2024) 22:961. doi: 10.1186/s12967-024-05737-z, PMID: 39438936 PMC11515775

[29] Gardiner C Weakley J Burke LM Roach GD Sargent C Maniar N. The effect of caffeine on subsequent sleep: a systematic review and meta-analysis. Sleep Med Rev. (2023) 69:101764. doi: 10.1016/j.smrv.2023.101764, PMID: 36870101

[30] Reichert CF Deboer T Landolt H. Adenosine, caffeine, and sleep–wake regulation: state of the science and perspectives. J Sleep Res. (2022) 31:e13597. doi: 10.1111/jsr.13597, PMID: 35575450 PMC9541543

[31] Marcus GM. Coffee’s effects on cardiac arrhythmias, physical activity, sleep and serum glucose: insights from the coffee and real-time atrial and ventricular ectopy trial. Clin Transl Med. (2023) 13:e1348. doi: 10.1002/ctm2.1348, PMID: 37501286 PMC10374881

[32] Pauchon B Beauchamps V Gomez-Mérino D Erblang M Drogou C Beers PV. Caffeine intake alters recovery sleep after sleep deprivation. Nutrients. (2024) 16:3442. doi: 10.3390/nu16203442, PMID: 39458438 PMC11510014

[33] Rains JC. Sleep and migraine: assessment and treatment of comorbid sleep disorders. Headache. (2018) 58:1074–91. doi: 10.1111/head.13357, PMID: 30095163

[34] Fenton RA Poulsen SB de la Mora Chavez S Soleimani M Busslinger M Dominguez Rieg JA. Caffeine-induced diuresis and natriuresis is independent of renal tubular NHE3. Am J Physiol Renal Physiol. (2015) 308:F1409–20. doi: 10.1152/ajprenal.00129.2015, PMID: 25925253 PMC4587593

[35] Reuter SE Schultz HB Ward MB Grant CL Paech GM Banks S. The effect of high-dose, short-term caffeine intake on the renal clearance of calcium, sodium and creatinine in healthy adults. Br J Clin Pharmacol. (2021) 87:4461–6. doi: 10.1111/bcp.14856, PMID: 33852164

[36] Barghouthy Y Corrales M Doizi S Somani BK Traxer O. Tea and coffee consumption and pathophysiology related to kidney stone formation: a systematic review. World J Urol. (2021) 39:2417–26. doi: 10.1007/s00345-020-03466-8, PMID: 33052484

