# Supplementary material for: The S-shaped association between dietary caffeine intake and severe headache or migraine: a cross-sectional study based on NHANES
Source: Front Neurol. 2025 May 14;16:1517942. doi: 10.3389/fneur.2025.1517942 (PMC12118965; doi:10.3389/fneur.2025.1517942)
Supplement: Supplementary file 1 [file Data_Sheet_1.docx]

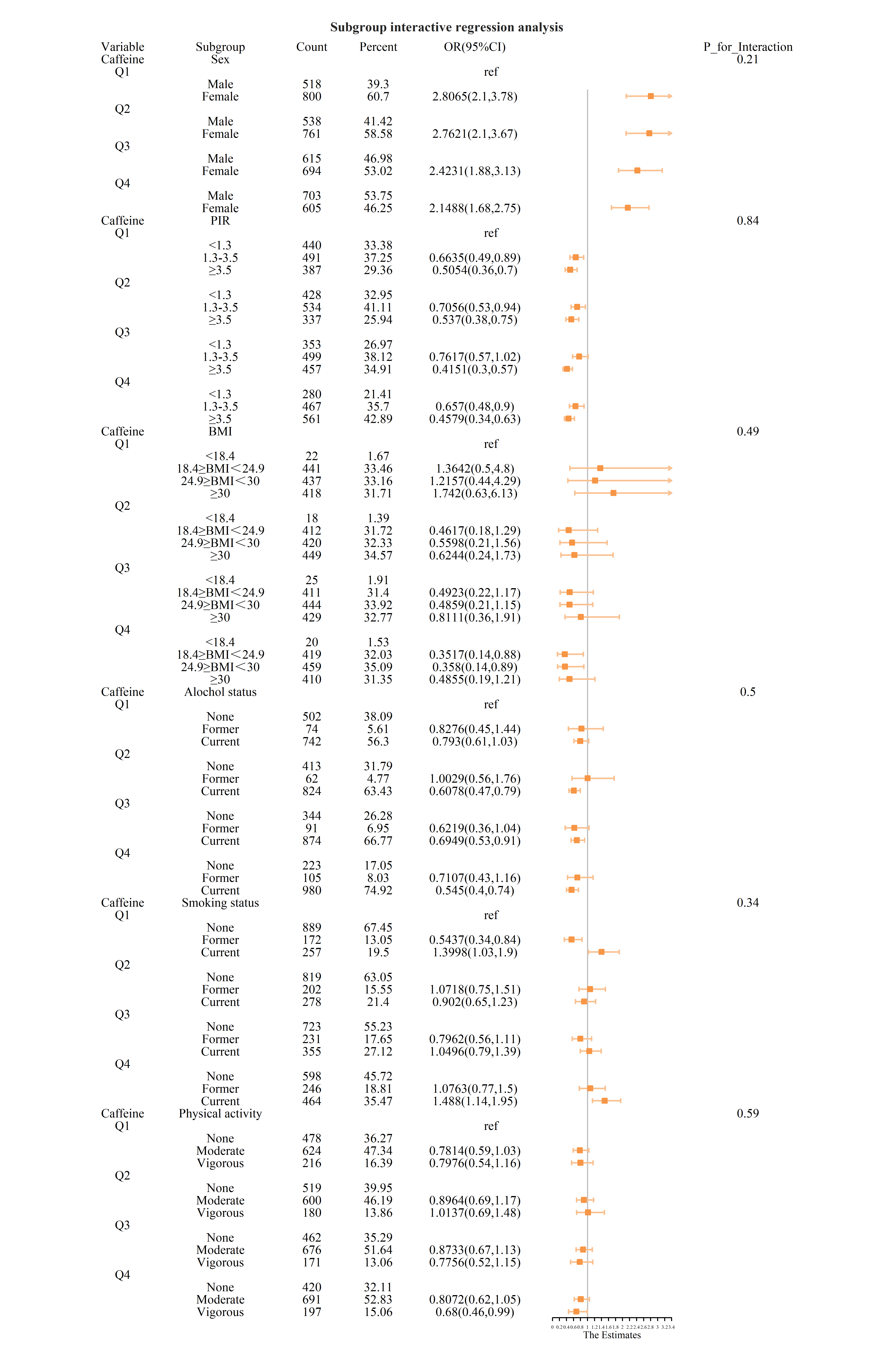
Supplementary Fig. 1. Subgroup interactive regression analysis of dietary caffeine and severe headache or migraine
